# Supplementary material for: Relative Efficacy of Weight Management, Exercise, and Combined Treatment for Muscle Mass and Physical Sarcopenia Indices in Adults with Overweight or Obesity and Osteoarthritis: A Network Meta-Analysis of Randomized Controlled Trials
Source: Nutrients. 2021 Jun 10;13(6):1992. doi: 10.3390/nu13061992 (PMC8230320; doi:10.3390/nu13061992)
Supplement: Supplementary file 1 [file nutrients-13-01992-s001.zip › nutrients-1200704-supplementary.pdf]

Supplementary table S1

Table S1. Database search formulas

Data base	Search terms for query (Final search date: May 12, 2021)
**Pubmed**	
#1	(elder adults) OR order adults
#2	((obesity) OR obese) OR overweight
#3	((osteoarthritis) OR gonarthritis) OR arthritis
#4	((((physical activity) OR exercise training) OR strengthening exercise) OR neuromuscular electrical stimulation) OR blood flow restriction
#5	((Diet intervention) OR weight loss) OR energy restriction
#6	(Randomized controlled trial) OR Randomization
#7	(((((#1) AND #2) AND #3) AND #4) AND #5) AND #6
**Physiotherapy Evidence Database (PEDro)**	
	Method: clinical trial
	Abstract & Title:
#1	elderly
#2	osteoarthritis
#3	overweight OR obesity
#4	diet
#5	physical activity
#6	exercise training
#7	neuromuscular electrical stimulation
#8	blood flow restriction
**Excerpta Medica dataBASE (EMBASE)**	
#1	'order adults '
#2	'osteoarthritis'
#3	'obesity' OR 'overweight'
#4	'diet'
#5	'weight loss'
#6	'exercise training'
#7	'physical activity'
#8	neuromuscular electrical stimulation
#9	blood flow restriction
#10	#1 AND #2 AND #3
#11	#4 OR #5 OR #6 OR #7 OR #8 OR #9
#12	#10 AND #11 AND ([randomized controlled trial]/lim) AND [humans]/lim

(continued)

Table S1. continued

Data base	Search terms for query
**Cochrane Library Database**	
#1	elderly
#2	osteoarthritis
#3	overweight OR obesity
#4	diet
#5	weight loss
#6	physical activity
#7	exercise training
#8	neuromuscular electrical stimulation
#9	blood flow restriction
#10	#1 AND #2 AND #3
#11	#4 OR #5 OR #6 OR #7 OR #8 OR #9
#12	Randomized controlled trial
#13	#10 AND #11 AND #12
**ClinicalKey database**	
#1	elderly OR older adults
#2	osteoarthritis
#3	overweight OR obesity
#4	diet OR weight loss
#5	exercise training
#6	randomized controlled trial
**China knowledge resource integrated database**	
#1	elderly
#2	osteoarthritis
#3	overweight OR obesity
#4	diet
#5	weight loss
#6	exercise training
#7	#4 OR #5 OR #6
#8	randomized controlled trial
#9	#1 AND #2 AND #3 AND #7 AND #8
**Google Scholar**	
#1	allintitle: elderly OR older adults
#2	allintitle: osteoarthritis
#3	allintitle: overweight OR obesity
#4	allintitle: diet OR weight loss
#5	allintitle: exercise training
#6	allintitle: randomized controlled trial

Table S2. Summary of methodological quality based on the PEDro classification scale[a]

Study author (year)	Overall[b]	Eligibility criteria[c]	1	2	3	4	5	6	7	8	9	10
Beavers, 2015	5/10[d]	*X*	*X*		*X*			*X*[d]			*X*	*X*
Christensen 2005	5/10	*X*	*X*		*X*				*X*		*X*	*X*
Christensen 2013	8/10	*X*	*X*	*X*	*X*			*X*	*X*	*X*	*X*	*X*
Christensen 2015	7/10[d]		*X*	*X*	*X*			*X*[d]		*X*	*X*	*X*
Christensen 2017	7/10	*X*	*X*	*X*	*X*			*X*		*X*	*X*	*X*
Ghroubi, 2008	4/10[d]	*X*	*X*		*X*						*X*[d]	*X*
Gill, 2009	7/10	*X*	*X*	*X*	*X*			*X*	*X*		*X*	*X*
Kuptniratsaikul, 2019	8/10	*X*	*X*	*X*	*X*			*X*	*X*	*X*	*X*	*X*
Lim 2010	7/10	*X*	*X*		*X*			*X*	*X*	*X*	*X*	*X*
López-Gómez, 2020	6/10	*X*	*X*		*X*				*X*	*X*	*X*	*X*
Magrans-Courtney, 2011	7/10	*X*	*X*		*X*			*X*	*X*	*X*	*X*	*X*
Mahmoud 2017	7/10	*X*	*X*		*X*			*X*	*X*	*X*	*X*	*X*
Mangani, 2006	5/10[d]	*X*	*X*		*X*			X[d]			*X*	*X*[d]
Matsuse, 2020	6/10		*X*	*X*	*X*			*X*			*X*	*X*
McLeod, 2020	5/10	*X*	*X*		*X*				*X*		*X*	*X*
Messier, 2000	6/10	*X*	*X*		*X*			*X*	*X*		*X*	*X*
Messier, 2013	7/10	*X*	*X*		*X*			*X*	*X*	*X*	*X*	*X*
Miller, 2006	5/10	*X*	*X*		*X*				*X*		*X*	*X*
Miller, 2012	5/10	*X*	*X*		*X*				*X*		*X*	*X*
Rabe, 2018	5/10	*X*	*X*		*X*			*X*			*X*	*X*
Robbins, 2020	8/10		*X*	*X*	*X*			*X*	*X*	*X*	*X*	*X*
Rosemffet, 2004	4/10		*X*		*X*						*X*	*X*
Segal, 2012a	7/10	*X*	*X*	*X*	*X*			*X*	*X*		*X*	*X*
Segal, 2012b	7/10	*X*	*X*	*X*	*X*			*X*	*X*		*X*	*X*
Skou, 2015	8/10	*X*	*X*	*X*	*X*			*X*	*X*	*X*	*X*	*X*
Skou, 2018	8/10	*X*	*X*	*X*	*X*			*X*	*X*	*X*	*X*	*X*
Swank, 2011	5/10	*X*	*X*		*X*				*X*		*X*	*X*
Tak, 2005	7/10	*X*	*X*		*X*			*X*	*X*	*X*	*X*	*X*
Talbot, 2003a	5/10	*X*	*X*		*X*				*X*		*X*	*X*
Talbot, 2003b	5/10	*X*	*X*		*X*				*X*		*X*	*X*
Toda, 2000	6/10	*X*	*X*		*X*				*X*	*X*	*X*	*X*
Toda, 2001	7/10	*X*	*X*		*X*			*X*	*X*	*X*	*X*	*X*
Wallis, 2017	8/10	*X*	*X*	*X*	*X*			*X*	*X*	*X*	*X*	*X*
Wang, 2007	5/10[d]	*X*	*X*		*X*				*X*[d]		*X*	*X*
Summary[e]		**30**	**34**	**12**	**34**	**0**	**0**	**22**	**26**	**16**	**34**	**34**

[a]PEDro, Physiotherapy Evidence Database. Guidline of PEDro scale is available from PEDro database (https://www.pedro.org.au/english/downloads/pedro-scale/).

[b]Points of methodological quality are denoted as "*X*" for fulfilled criteria.

[c]This item is not used to calculate the total score.

[d]Score was determined by a third assessor.

[e]This was calculated as the number of studies satisfied.

PEDro classification scale: 1 = random allocation, 2 = concealed allocation, 3 = similarity at the baseline, 4 = subject blinding, 5 = therapist blinding, 6 = assessor blinding, 7 = more than 85% follow-up for at least one key outcome, 8 = intention-to-treat analysis, 9 = between-group statistical comparison for at least one key outcome, 10 = point and variability measures for at least one key outcome. Methodological quality: high, ≥7 points; medium, 4–6 points; low, ≤3 points.

Table S3. League table for pairwise and network meta-analysis of mean change in muscle mass from baseline

Relative effects of network meta-analyses	Direct evidence of pairwise meta-analyses																	
	V1	V2	V3	V4	V5	V6	V7	V8	V9	V10	V11	V12	V13	V14	V15	V16	V17	V18
1	**RMMR + RET**				0.48 (-0.07, 1.03)				0.64 (0.01, 1.29)			0.61 (-0.06, 1.28)	0.76 (0.19, 1.33)					1.26 (0.59, 1.92)
2	0.41 (-0.24, 1.06)	**RMMR_DIA + MET**				0.15 (-0.23, 0.54)								0.36 (-0.03, 0.75)				1.16 (0.75, 1.58)
3	0.36 (-0.53, 1.26)	-0.05 (-0.92, 0.83)	**AQET**				0.38 (-0.39, 1.15)		0.27 (-0.48, 1.03)									.
4	0.39 (-0.59, 1.38)	-0.02 (-1.01, 0.98)	0.03 (-1.03, 1.10)	**RET + BFR**					0.37 (-0.42, 1.16)									.
5	0.47 (-0.08, 1.02)	0.06 (-0.60, 0.71)	0.11 (-0.80, 1.01)	0.07 (-0.92, 1.07)	**RMMR + AET**				0.55 (-0.13, 1.23)			0.52 (-0.18, 1.23)	0.34 (-0.22, 0.91)					1.17 (0.47, 1.86)
6	0.60 (-0.07, 1.28)	0.19 (-0.15, 0.54)	0.24 (-0.65, 1.14)	0.21 (-0.80, 1.22)	0.14 (-0.54, 0.82)	**RMMR_DIA**								0.21 (-0.18, 0.60)				1.02 (0.35, 1.69)
7	0.60 (-0.17, 1.38)	0.19 (-0.52, 0.90)	0.24 (-0.49, 0.97)	0.21 (-0.79, 1.21)	0.14 (-0.65, 0.92)	-0.00 (-0.74, 0.74)	**IMET**		-0.11 (-0.88, 0.66)									1.13 (0.27, 1.98)
8	0.71 (-0.15, 1.57)	0.30 (-0.37, 0.96)	0.34 (-0.70, 1.39)	0.31 (-0.83, 1.46)	0.24 (-0.63, 1.11)	0.10 (-0.57, 0.78)	0.10 (-0.81, 1.02)	**DIA + MET**						0.26 (-0.32, 0.84)				
9	0.77 (0.17, 1.36)	0.35 (-0.25, 0.96)	0.40 (-0.32, 1.12)	0.37 (-0.42, 1.16)	0.30 (-0.31, 0.90)	0.16 (-0.47, 0.80)	0.16 (-0.46, 0.78)	0.06 (-0.78, 0.89)	**RET**		0.06 (-0.80, 0.93)	-0.03 (-0.68, 0.61)	-0.25 (-0.91, 0.42)					0.61 (-0.01, 1.24)
10	0.81 (-0.01, 1.62)	0.40 (-0.44, 1.23)	0.44 (-0.62, 1.51)	0.41 (-0.73, 1.56)	0.34 (-0.47, 1.15)	0.20 (-0.65, 1.06)	0.20 (-0.75, 1.16)	0.10 (-0.91, 1.11)	0.04 (-0.79, 0.87)	**RUMR**			0.08 (-0.52, 0.69)					
11	0.83 (-0.22, 1.88)	0.42 (-0.64, 1.47)	0.47 (-0.65, 1.59)	0.44 (-0.73, 1.60)	0.36 (-0.69, 1.42)	0.23 (-0.85, 1.30)	0.23 (-0.83, 1.29)	0.12 (-1.08, 1.32)	0.06 (-0.80, 0.93)	0.02 (-1.17, 1.22)	**DIA + RET**							
12	0.82 (0.19, 1.46)	0.41 (-0.27, 1.09)	0.46 (-0.46, 1.38)	0.43 (-0.58, 1.44)	0.36 (-0.29, 1.00)	0.22 (-0.48, 0.92)	0.22 (-0.58, 1.02)	0.12 (-0.77, 1.00)	0.06 (-0.57, 0.69)	0.02 (-0.85, 0.88)	-0.01 (-1.07, 1.06)	**AET**	-0.22 (-0.90, 0.47)					0.64 (-0.01, 1.29)
13	0.89 (0.35, 1.43)	0.48 (-0.09, 1.05)	0.53 (-0.35, 1.40)	0.50 (-0.48, 1.47)	0.42 (-0.12, 0.97)	0.29 (-0.32, 0.89)	0.29 (-0.45, 1.02)	0.18 (-0.62, 0.99)	0.12 (-0.44, 0.69)	0.08 (-0.52, 0.69)	0.06 (-0.97, 1.09)	0.07 (-0.55, 0.68)	**RMMR**	-0.32 (-1.29, 0.65)		0.05 (-0.88, 0.99)		0.52 (-0.03, 1.07)
14	0.97 (0.34, 1.60)	0.56 (0.24, 0.88)	0.61 (-0.26, 1.47)	0.57 (-0.41, 1.56)	0.50 (-0.14, 1.14)	0.36 (0.02, 0.71)	0.37 (-0.34, 1.07)	0.26 (-0.32, 0.84)	0.20 (-0.39, 0.80)	0.16 (-0.66, 0.98)	0.14 (-0.91, 1.19)	0.15 (-0.52, 0.81)	0.08 (-0.47, 0.63)	**MET**		0.38 (-0.60, 1.35)	0.29 (-0.13, 0.70)	0.15 (-0.24, 0.53)
15	1.26 (0.37, 2.16)	0.85 (0.12, 1.59)	0.90 (-0.17, 1.97)	0.87 (-0.30, 2.04)	0.80 (-0.11, 1.70)	0.66 (-0.09, 1.41)	0.66 (-0.28, 1.60)	0.56 (-0.35, 1.46)	0.50 (-0.37, 1.37)	0.46 (-0.58, 1.50)	0.43 (-0.79, 1.66)	0.44 (-0.48, 1.36)	0.37 (-0.47, 1.22)	0.30 (-0.40, 0.99)	**IMMR_DIA**		0.13 (-0.44, 0.71)	
16	1.29 (0.36, 2.21)	0.88 (0.04, 1.71)	0.92 (-0.19, 2.04)	0.89 (-0.32, 2.10)	0.82 (-0.11, 1.75)	0.68 (-0.17, 1.54)	0.68 (-0.32, 1.69)	0.58 (-0.42, 1.58)	0.52 (-0.39, 1.44)	0.48 (-0.55, 1.51)	0.46 (-0.80, 1.71)	0.46 (-0.49, 1.42)	0.40 (-0.44, 1.23)	0.32 (-0.50, 1.13)	0.02 (-1.02, 1.07)	**RMMR + MET**		-0.21 (-1.16, 0.74)
17	1.40 (0.71, 2.09)	0.99 (0.53, 1.44)	1.04 (0.13, 1.94)	1.00 (-0.02, 2.03)	0.93 (0.23, 1.63)	0.79 (0.31, 1.28)	0.80 (0.05, 1.55)	0.69 (-0.01, 1.39)	0.63 (-0.02, 1.29)	0.59 (-0.27, 1.46)	0.57 (-0.51, 1.65)	0.58 (-0.14, 1.29)	0.51 (-0.11, 1.13)	0.43 (0.04, 0.82)	0.13 (-0.44, 0.71)	0.11 (-0.76, 0.98)	**RUMR_DIA**	-0.15 (-0.56, 0.27)
18	1.40 (0.83, 1.97)	0.99 (0.67, 1.30)	1.04 (0.21, 1.86)	1.00 (0.06, 1.95)	0.93 (0.35, 1.51)	0.79 (0.43, 1.16)	0.79 (0.15, 1.44)	0.69 (0.03, 1.35)	0.63 (0.11, 1.16)	0.59 (-0.19, 1.37)	0.57 (-0.44, 1.58)	0.58 (-0.03, 1.18)	0.51 (0.01, 1.00)	0.43 (0.13, 0.73)	0.13 (-0.56, 0.83)	0.11 (-0.69, 0.91)	-0.00 (-0.39, 0.39)	**UC**

Pairwise (upper right portion) and network (lower left portion) meta-analysis results are presented for mean change (from baseline) in muscle mass outcomes. Interventions are reported in order of efficacy ranked according to P scores. Effect estimation is presented in standardized mean difference (SMD) with 95% CI. Significant results are marked in red.

AET, aerobic exercise training; AQET, aquatic exercise training; BFR, blood flow restriction; DIA, diet instruction and advisement; IMET, isometric exercise training; IMMR, intermittent multi-meal replacement; MET, multicomponent exercise training; RUMR, regular uni-meal replacement; RMMR, regular multi-meal replacement; RET, resistance exercise training; UC, usual care.

Table S4. League table for pairwise and network meta-analysis of mean change in muscle strength from baseline

Relative effects of network meta-analyses	Direct evidence of pairwise meta-analyses									
	V1	V2	V3	V4	V5	V6	V7	V8	V9	V10
1	**RMMR_DIA + MET**			0.55 (0.22, 0.87)						1.64 (1.29, 1.99)
2	0.44 (0.01, 0.88)	**DIA + MET**		0.17 (-0.24, 0.59)			0.52 (0.26, 0.77)	0.68 (0.26, 1.11)		
3	0.42 (-0.15, 0.98)	-0.02 (-0.50, 0.45)	**AET**				0.51 (0.10, 0.91)		0.59 (0.17, 1.01)	
4	0.55 (0.22, 0.87)	0.10 (-0.19, 0.40)	0.13 (-0.34, 0.59)	**MET**	0.02 (-0.27, 0.30)		0.42 (0.20, 0.65)	0.51 (0.10, 0.93)		1.09 (0.76, 1.43)
5	0.62 (0.20, 1.04)	0.18 (-0.17, 0.53)	0.20 (-0.29, 0.69)	0.07 (-0.20, 0.35)	**RUMR_DIA**		0.25 (-0.04, 0.53)			
6	0.70 (0.14, 1.25)	0.25 (-0.21, 0.71)	0.28 (-0.29, 0.84)	0.15 (-0.30, 0.60)	0.07 (-0.40, 0.55)	**RMMR + MET**	0.23 (-0.16, 0.62)			
7	0.93 (0.53, 1.32)	0.48 (0.24, 0.73)	0.51 (0.10, 0.91)	0.38 (0.16, 0.60)	0.30 (0.03, 0.58)	0.23 (-0.16, 0.62)	**UC**	-0.19 (-0.59, 0.22)	0.08 (-0.31, 0.48)	
8	0.96 (0.47, 1.45)	0.51 (0.14, 0.89)	0.54 (0.00, 1.08)	0.41 (0.04, 0.78)	0.34 (-0.09, 0.76)	0.26 (-0.26, 0.79)	0.03 (-0.32, 0.39)	**DIA**		
9	1.01 (0.45, 1.56)	0.57 (0.10, 1.03)	0.59 (0.17, 1.01)	0.46 (0.01, 0.91)	0.39 (-0.09, 0.87)	0.31 (-0.24, 0.87)	0.08 (-0.31, 0.48)	0.05 (-0.48, 0.58)	**RET**	
10	1.64 (1.29, 1.99)	1.20 (0.75, 1.65)	1.22 (0.65, 1.80)	1.09 (0.76, 1.43)	1.02 (0.59, 1.45)	0.95 (0.39, 1.50)	0.72 (0.32, 1.12)	0.68 (0.19, 1.18)	0.63 (0.07, 1.20)	**RMMR_DIA**

Pairwise (upper right portion) and network (lower left portion) meta-analysis results are presented for mean change (from baseline) in muscle mass outcomes. Interventions are reported in order of efficacy ranked according to P scores. Effect estimation is presented in standardized mean difference (SMD) with 95% CI. Significant results are marked in red.

AET, aerobic exercise training; DIA, diet instruction and advisement; MET, multicomponent exercise training; RUMR, regular uni-meal replacement; RMMR, regular multi-meal replacement; RET, resistance exercise training; UC, usual care.

Table S5. League table for pairwise and network meta-analysis of mean change in walk speed from baseline

Relative effects of network meta-analyses	Direct evidence of pairwise meta-analyses								
	V1	V2	V3	V4	V5	V6	V7	V8	V9
1	**AET**		0.00 (-0.91, 0.91)				0.41 (-0.04, 0.85)		0.47 (0.15, 0.80)
2	0.01 (-0.44, 0.46)	**DIA + MET**						1.12 (0.21, 2.03)	0.36 (0.02, 0.69)
3	0.00 (-0.91, 0.91)	-0.01 (-1.03, 1.01)	**NMES + AET**						
4	0.18 (-0.48, 0.84)	0.17 (-0.55, 0.88)	0.18 (-0.95, 1.30)	**AQET**			0.22 (-0.30, 0.74)		
5	0.18 (-0.42, 0.78)	0.17 (-0.46, 0.80)	0.18 (-0.91, 1.27)	0.00 (-0.75, 0.76)	**NMES**		0.38 (-0.34, 1.10)		0.11 (-0.61, 0.83)
6	0.23 (-0.30, 0.75)	0.22 (-0.30, 0.74)	0.23 (-0.82, 1.28)	0.05 (-0.71, 0.82)	0.05 (-0.63, 0.73)	**RMMR + MET**			0.23 (-0.19, 0.65)
7	0.40 (0.00, 0.80)	0.39 (-0.10, 0.88)	0.40 (-0.60, 1.39)	0.22 (-0.30, 0.74)	0.22 (-0.32, 0.76)	0.17 (-0.39, 0.73)	**RET**		0.15 (-0.27, 0.57)
8	0.44 (-0.11, 0.99)	0.43 (-0.07, 0.94)	0.44 (-0.62, 1.51)	0.27 (-0.52, 1.05)	0.26 (-0.44, 0.97)	0.22 (-0.40, 0.83)	0.05 (-0.54, 0.63)	**MET**	0.22 (-0.28, 0.73)
9	0.46 (0.14, 0.78)	0.45 (0.13, 0.77)	0.46 (-0.51, 1.43)	0.28 (-0.36, 0.92)	0.28 (-0.26, 0.82)	0.23 (-0.19, 0.65)	0.06 (-0.31, 0.43)	0.01 (-0.43, 0.46)	**UC**

Pairwise (upper right portion) and network (lower left portion) meta-analysis results are presented for mean change (from baseline) in walking speed outcomes. Interventions are reported in order of efficacy ranked according to P scores. Effect estimation is presented in standardized mean difference (SMD) with 95% CI. Significant results are marked in red.

AET, aerobic exercise training; AQET, aquatic exercise training; DIA, diet instruction and advisement; NMES, neuromuscular electric stimulation; MET, multicomponent exercise training; RMMR, regular multi-meal replacement; RET, resistance exercise training; UC, usual care.

Table S6. Results of network meta–regression analyses for associations of moderators with treatment efficiency

	Muscle mass				Muscle strength				Walk speed			
Moderator	B[a]	SE	Median	95% Credibility Interval	B[a]	SE	Median	95% Credibility Interval	B[a]	SE	Median	95% Credibility Interval
Age	–0.276	0.0016	0.275	–0.372, 0.923	–0.211	0.0049	–0.240	–2.165, 1.852	–0.643	0.0040	-0.529	–2.478, 0.681
BMI	–0.381	0.0015	–0.386	–0.979, 0.245	0.650	0.0064	–2.002	0.663, 3.178	0.641	0.0030	0.591	–0.414, 1.934
Sex distribution[b]	–0.159	0.0016	–0.162	–0.768, 0.479	–0.232	0.0129	–0.181	–5.691, 5.025	0.263	0.0018	0.254	–0.413, 0.986
Treatment duration	–0.324	0.0018	–0.332	–1.001, 0.390	–1.357	0.0064	–1.428	–0.332, 1.411	0.142	0.0022	0.142	–0.308, 0.594
Sample size	–0.491	0.00267	–0.507	–1.497, 0.591	–0.339	0.0044	–0.339	–2.167, 1.454	1.633	0.0028	0.158	–0.443, 0.810
PEDro score	–0.172	0.0014	–0.182	–0.694, 0.391	–0.558	0.0054	–0.575	–2.748, 1.629	0.210	0.0033	0.203	–0.401, 0.862

[a]Data represents the change in effects associated with the moderator indicated. SE, standard error.

[b]The proportion of female participants in sample

Table S7. Summary of adverse events of the included studies.

Study author (year)	Diet		Exercise	
	Related to treatment	Not related to treatment	Related to treatment	Not related to treatment
**Diet alone**				
Christensen 2005	3 (48)	0		
Christensen 2017	29 (153)	35 (153)		
López-Gómez, 2020	NR	NR		
**Exercise alone**				
Gill, 2009			0	12 (82)
Kuptniratsaikul, 2019			18 (71)	6 (71)
Lim 2010			3 (75)	1 (75)
Mahmoud 2017			NR	NR
Mangani, 2006			NR	NR
Matsuse, 2020			3 (28)	1 (28)
Rabe, 2018			0	4 (42)
Rosemffet, 2004			NR	NR
Segal, 2012a			NR	NR
Segal, 2012b			NR	NR
Swank, 2011			NR	NR
Tak, 2005			NR	NR
Talbot, 2003a			NR	NR
Talbot, 2003b			NR	NR
Wallis, 2017			2 (23)	0
**Combined treatment**				
Beavers, 2015	NR	NR	NR	NR
Christensen 2013	0	0	1 (58)	0
Christensen 2015	0	0	1 (58)	0
Ghroubi, 2008	NR	NR	NR	NR
Magrans-Courtney, 2011	NR	NR	NR	NR
McLeod, 2020	NR	NR	NR	NR
Messier, 2000	NR	NR	NR	NR
Messier, 2013	0	1 (152)	3 (152)	3 (150)
Miller, 2006	0	0	0	0
Miller, 2012	0	0	0	0
Robbins, 2020	6 (37)	0	7 (57)	0
Skou, 2015	0	0	26 (50)	12 (50)
Skou, 2018	NR	NR	NR	NR
Toda, 2000	NR	NR	NR	NR
Toda, 2001	NR	NR	NR	NR
Wang, 2007	0	0	0	0

All data are presented as number of patients (total sample size of the treatment group).
NR, not reported.

Supplementary figure S1

(A) Overall follow-up duration

Treatment	Direct Comparisons	SMD	95% CI	P−score
RMMR + RET	1	1.40	[0.83; 1.97]	0.95
RMMR_DIA + MET	3	0.99	[0.67; 1.30]	0.78
AQET	0	1.04	[0.21; 1.86]	0.77
RET + BFR	0	1.00	[0.06; 1.95]	0.73
RMMR + AET	1	0.93	[0.35; 1.51]	0.72
RMMR_DIA	1	0.79	[0.43; 1.16]	0.63
IMET	1	0.79	[0.15; 1.44]	0.62
DIA + MET	0	0.69	[0.03; 1.35]	0.55
RET	1	0.63	[0.11; 1.16]	0.50
RUMR	0	0.59	[−0.19; 1.37]	0.48
DIA + RET	0	0.57	[−0.44; 1.58]	0.46
AET	1	0.58	[−0.03; 1.18]	0.45
RMMR	2	0.51	[0.01; 1.00]	0.41
MET	3	0.43	[0.13; 0.73]	0.35
IMMR_DIA	0	0.13	[−0.56; 0.83]	0.20
RMMR + MET	1	0.11	[−0.69; 0.91]	0.19
RUMR_DIA	2	−0.00	[−0.39; 0.39]	0.10

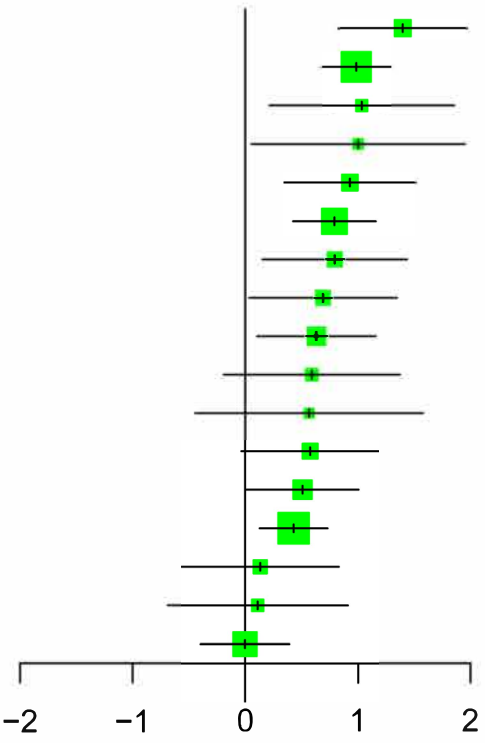

(B) Follow up >6 months

Treatment	Direct Comparisons	SMD	95% CI	P−score
RMMR_DIA + MET	0	0.50	[0.11; 0.90]	0.97
RMMR_DIA	0	0.35	[−0.05; 0.75]	0.78
MET	2	0.14	[−0.16; 0.44]	0.51
IMMR_DIA	0	−0.01	[−0.51; 0.50]	0.33
RUMR_DIA	2	−0.14	[−0.45; 0.16]	0.09

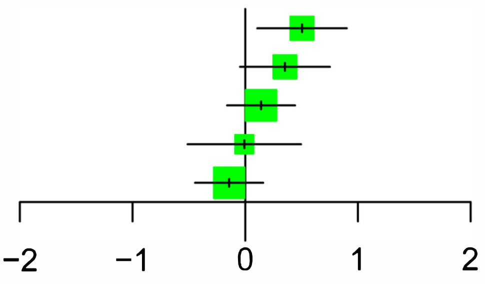

(C) Follow up >3 months, ≤6 months

Treatment	Direct Comparisons	SMD	95% CI	P−score
RMMR_DIA + MET	3	1.16	[0.86; 1.47]	NA

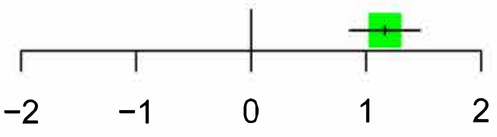

(D) Follow up ≤3 months

Treatment	Direct Comparisons	SMD	95% CI	P−score
RMMR + RET	1	1.44	[0.57; 2.31]	0.88
AQET	0	1.05	[−0.17; 2.27]	0.69
RMMR_DIA	1	1.02	[−0.03; 2.07]	0.67
RET + BFR	0	1.01	[−0.39; 2.42]	0.66
RMMR + AET	1	0.89	[0.02; 1.77]	0.63
IMET	1	0.82	[−0.11; 1.74]	0.57
DIA + MET	0	0.73	[−0.81; 2.27]	0.53
RET	1	0.64	[−0.19; 1.48]	0.47
DIA + RET	0	0.58	[−0.87; 2.03]	0.45
AET	1	0.56	[−0.40; 1.52]	0.43
RUMR	0	0.53	[−0.73; 1.79]	0.42
MET	1	0.47	[−0.70; 1.64]	0.40
RMMR	2	0.45	[−0.30; 1.20]	0.36
RMMR + MET	1	0.10	[−1.04; 1.24]	0.22

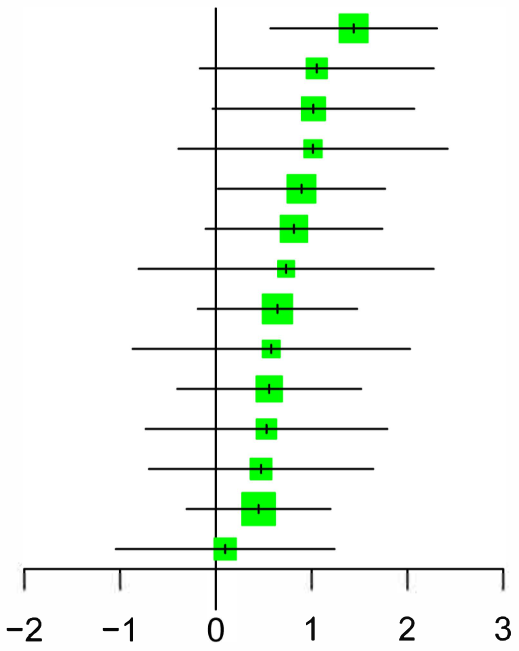

Figure S1. Forest plot summarizing effects of diet, exercise interventions, and combined treatments on changes of muscle mass at each follow up duration. Each point estimate (square) at each time frame and during an overall duration presents the network combined effect (standard mean difference) of the muscle mass with reference to UC, with 95% CI (horizontal line). Results plotted on the right−hand side indicate effects in favor of treatment.
95% CI = 95% confidence interval; AET, aerobic exercise training; AQET, aquatic exercise training; BFR, blood flow restriction; DIA, diet instruction and advisement; IMET, isometric exercise training; IMMR, intermittent multi−meal replacement; MET, multicomponent exercise training; RET, resistance exercise training; RMMR, regular multi−meal replacement; RUMR, regular uni−meal replacement; Std = standard; UC, usual care.

Supplementary figure S2

(A) Overall follow-up duration

Treatment	Direct Comparisons	SMD	95% CI	P−score
RET	1	1.93	[0.62; 3.24]	0.82
RET_BFR	0	2.03	[0.27; 3.78]	0.81
NMES + MET	0	1.27	[−0.61; 3.16]	0.61
AET	1	1.23	[−0.46; 2.93]	0.60
NMES	1	1.18	[0.00; 2.37]	0.59
AQET	0	1.23	[−1.08; 3.53]	0.59
MET	1	0.97	[−0.35; 2.29]	0.52
IMET	1	0.87	[−0.80; 2.54]	0.48
RMMR + MET	2	0.65	[−0.48; 1.79]	0.41
DIA + MET	0	0.55	[−1.62; 2.72]	0.38
RMMR_DIA + MET	1	0.41	[−1.18; 2.00]	0.34
RMMR	1	−0.11	[−1.66; 1.45]	0.17

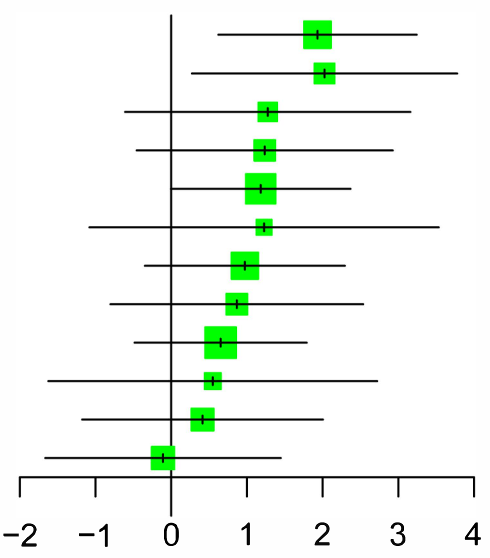

(B) Follow up >6 months

Treatment	Direct Comparisons	SMD	95% CI	P−score
RMMR + MET	1	0.15	[−0.17; 0.46]	0.18

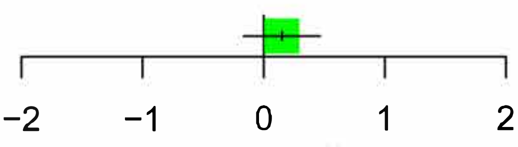

(C) Follow up >3 months, ≤6 months

Treatment	Direct Comparisons	SMD	95% CI	P−score
NMES	1	0.71	[0.01; 1.41]	0.79
AET	1	0.70	[0.01; 1.40]	0.78
RMMR_DIA + MET	1	0.41	[−0.05; 0.88]	0.57
RMMR + MET	1	0.15	[−0.17; 0.46]	0.29

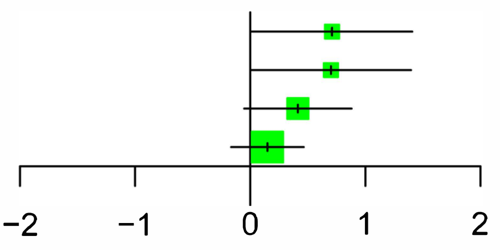

(D) Follow up ≤3 months

Treatment	Direct Comparisons	SMD	95% CI	P−score
RET_BFR	0	2.06	[0.28; 3.84]	0.79
RET	1	1.97	[0.64; 3.30]	0.78
NMES + MET	0	1.42	[−0.52; 3.35]	0.60
RMMR + MET	1	1.23	[−0.46; 2.93]	0.55
NMES	1	1.26	[0.04; 2.47]	0.55
AET	1	1.23	[−0.48; 2.95]	0.54
AQET	0	1.23	[−1.12; 3.57]	0.54
MET	1	1.18	[−0.23; 2.59]	0.53
IMET	1	0.87	[−0.83; 2.56]	0.42
DIA + MET	0	0.76	[−1.49; 3.00]	0.39
RMMR	1	0.14	[−1.52; 1.81]	0.20

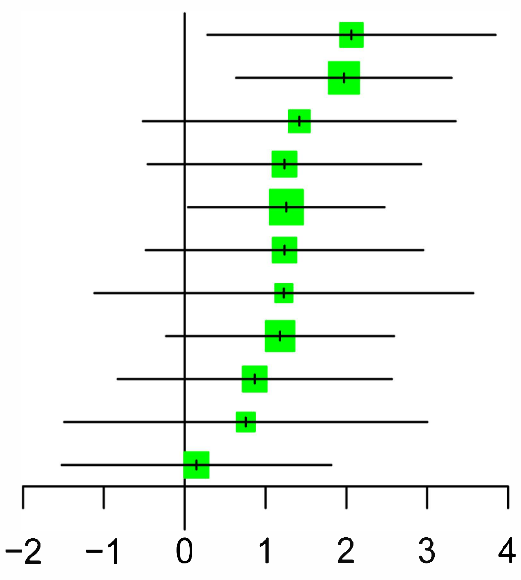

Figure S2. Forest plot summarizing effects of diet, exercise interventions, and combined treatments on changes of muscle strength at each follow up duration. Each point estimate (square) at each time frame and during an overall duration presents the network combined effect (standard mean difference) of the muscle mass with reference to UC, with 95% CI (horizontal line). Results plotted on the right−hand side indicate effects in favor of treatment.

95% CI = 95% confidence interval; AET, aerobic exercise training; DIA, diet instruction and advisement; MET, multicomponent exercise training; RET, resistance exercise training; RMMR, regular multi−meal replacement; RUMR, regular uni−meal replacement; Std = standard; UC, usual care.

Supplementary figure S3

(A) Overall follow-up duration

Treatment	Direct Comparisons	SMD	95% CI	P−score
AET	3	0.46	[0.14; 0.78]	0.23
DIA + MET	2	0.45	[0.13; 0.77]	0.25
NMES + AET	0	0.46	[−0.51; 1.43]	0.34
AQET	0	0.28	[−0.36; 0.92]	0.45
NMES	1	0.28	[−0.26; 0.82]	0.45
RMMR + MET	1	0.23	[−0.19; 0.65]	0.50
RET	1	0.06	[−0.31; 0.43]	0.73
MET	1	0.01	[−0.43; 0.46]	0.75

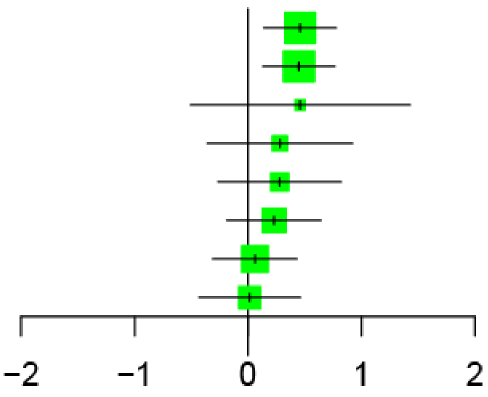

(B) Follow up >6 months

Treatment	Direct Comparisons	SMD	95% CI	P−score
AET	1	0.51	[0.16; 0.86]	0.08
DIA + MET	2	0.32	[0.04; 0.60]	0.30
RMMR + MET	1	0.23	[−0.10; 0.56]	0.43
RET	1	−0.08	[−0.41; 0.25]	0.89

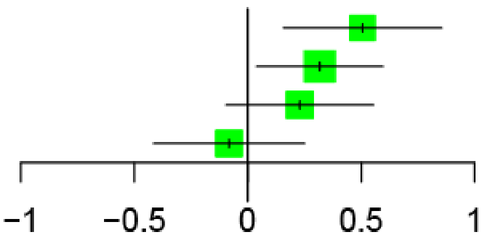

(C) Follow up >3 months, ≤6 months

Treatment	Direct Comparisons	SMD	95% CI	P−score
AET	2	0.55	[0.05; 1.05]	0.18
DIA + MET	1	0.55	[−0.01; 1.12]	0.19
AQET	0	0.36	[−0.55; 1.27]	0.37
RET	1	0.15	[−0.43; 0.72]	0.56
RMMR + MET	1	0.09	[−0.50; 0.69]	0.59
MET	1	−0.02	[−0.60; 0.57]	0.69
NMES	1	−0.12	[−0.96; 0.73]	0.73

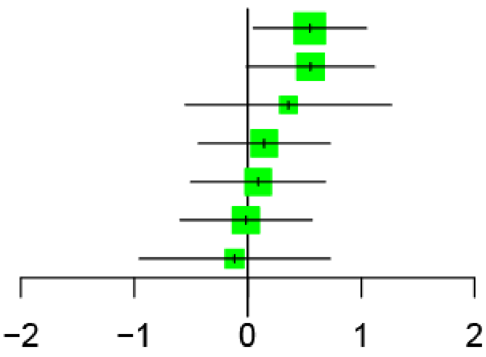

(D) Follow up ≤3 months

Treatment	Direct Comparisons	SMD	95% CI	P−score
AET	3	0.52	[0.25; 0.80]	0.13
NMES + AET	0	0.52	[−0.40; 1.44]	0.24
DIA + MET	1	0.36	[0.00; 0.72]	0.28
NMES	1	0.18	[−0.32; 0.68]	0.48
AQET	0	0.09	[−0.46; 0.64]	0.57
MET	1	−0.04	[−0.43; 0.35]	0.74
RET	1	−0.13	[−0.44; 0.17]	0.86

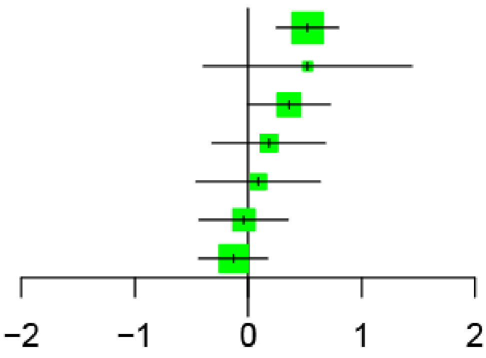

Figure S3. Forest plot summarizing effects of diet, exercise interventions, and combined treatments on changes of walk speed at each follow up duration. Each point estimate (square) at each time frame and during an overall duration presents the network combined effect (standard mean difference) of the muscle mass with reference to UC, with 95% CI (horizontal line). Results plotted on the right-hand side indicate effects in favor of treatment.
95% CI = 95% confidence interval; AET, aerobic exercise training; AQET, aquatic exercise training; DIA, diet instruction and advisement; IMET, isometric exercise training; MET, multicomponent exercise training; NMES, neuromuscular electric stimulation; RET, resistance exercise training; RMMR, regular multi−meal replacement; RUMR, regular uni−meal replacement; Std = standard; UC, usual care.

Supplementary figure S4

Comparison	Number of Studies	Direct Evidence	SMD	95%CI
**AET vs RET**				
Direct estimate	1	0.95	0.03	[−0.62; 0.68]
Indirect estimate			−1.32	[−4.10; 1.46]
Network estimate			−0.04	[−0.67; 0.59]
**AET vs RMMR**				
Direct estimate	1	0.88	−0.22	[−0.91; 0.47]
Indirect estimate			0.87	[−1.03; 2.78]
Network estimate			−0.09	[−0.74; 0.56]
**AET vs RMMR + AET**				
Direct estimate	1	0.85	−0.52	[−1.23; 0.18]
Indirect estimate			0.38	[−1.32; 2.08]
Network estimate			−0.39	[−1.04; 0.26]
**AET vs RMMR + RET**				
Direct estimate	1	0.90	−0.61	[−1.29; 0.06]
Indirect estimate			−2.90	[−4.89; −0.92]
Network estimate			−0.85	[−1.49; −0.21]
**AET vs UC**				
Direct estimate	1	0.95	0.64	[−0.01; 1.30]
Indirect estimate			2.02	[−0.72; 4.75]
Network estimate			0.72	[0.08; 1.35]
**AQET vs IMET**				
Direct estimate	1	0.91	0.38	[−0.39; 1.15]
Indirect estimate			−0.80	[−3.18; 1.58]
Network estimate			0.27	[−0.46; 1.00]
**AQET vs RET**				
Direct estimate	1	0.91	0.27	[−0.48; 1.03]
Indirect estimate			1.49	[−0.98; 3.97]
Network estimate			0.38	[−0.35; 1.10]
**IMET vs RET**				
Direct estimate	1	0.65	−0.11	[−0.88; 0.67]
Indirect estimate			0.51	[−0.55; 1.57]
Network estimate			0.11	[−0.52; 0.73]
**IMET vs UC**				
Direct estimate	1	0.57	1.13	[0.27; 1.98]
Indirect estimate			0.51	[−0.49; 1.50]
Network estimate			0.86	[0.21; 1.51]
**MET vs RMMR_DIA**				
Direct estimate	2	0.79	−0.21	[−0.60; 0.18]
Indirect estimate			−0.99	[−1.75; −0.23]
Network estimate			−0.37	[−0.72; −0.03]
**MET vs RMMR_DIA + MET**				
Direct estimate	2	0.69	−0.36	[−0.75; 0.03]
Indirect estimate			−1.03	[−1.62; −0.45]
Network estimate			−0.57	[−0.90; −0.25]
**MET vs RUMR_DIA**				
Direct estimate	2	0.91	0.27	[−0.14; 0.68]
Indirect estimate			1.71	[0.42; 2.99]
Network estimate			0.41	[0.01; 0.80]
**MET vs UC**				
Direct estimate	2	0.59	0.14	[−0.27; 0.55]
Indirect estimate			0.79	[0.30; 1.29]
Network estimate			0.41	[0.09; 0.72]
**RET vs RMMR**				
Direct estimate	1	0.83	−0.25	[−0.91; 0.42]
Indirect estimate			0.90	[−0.57; 2.37]
Network estimate			−0.05	[−0.66; 0.56]
**RET vs RMMR + AET**				
Direct estimate	1	0.80	−0.55	[−1.24; 0.13]
Indirect estimate			0.46	[−0.90; 1.83]
Network estimate			−0.35	[−0.96; 0.26]
**RET vs RMMR + RET**				
Direct estimate	1	0.84	−0.64	[−1.30; 0.01]
Indirect estimate			−1.70	[−3.20; −0.20]
Network estimate			−0.81	[−1.41; −0.21]
**RET vs UC**				
Direct estimate	1	0.77	0.61	[−0.01; 1.24]
Indirect estimate			1.24	[0.09; 2.39]
Network estimate			0.76	[0.21; 1.31]
**RMMR vs RMMR + AET**				
Direct estimate	2	1.00	−0.34	[−0.91; 0.22]
Indirect estimate			8.12	[0.08; 16.16]
Network estimate			−0.30	[−0.87; 0.27]
**RMMR vs UC**				
Direct estimate	1	0.83	0.86	[0.18; 1.54]
Indirect estimate			0.55	[−0.95; 2.05]
Network estimate			0.81	[0.19; 1.42]
**RMMR + AET vs RMMR + RET**				
Direct estimate	2	0.99	−0.48	[−1.04; 0.07]
Indirect estimate			3.39	[−3.69; 10.48]
Network estimate			−0.46	[−1.01; 0.09]
**RMMR + AET vs UC**				
Direct estimate	1	0.80	1.17	[0.47; 1.87]
Indirect estimate			0.87	[−0.51; 2.25]
Network estimate			1.11	[0.48; 1.73]
**RMMR + RET vs UC**				
Direct estimate	1	0.84	1.26	[0.59; 1.92]
Indirect estimate			3.16	[1.65; 4.67]
Network estimate			1.57	[0.96; 2.18]
**RMMR_DIA vs RMMR_DIA + MET**				
Direct estimate	2	0.79	−0.15	[−0.54; 0.24]
Indirect estimate			−0.36	[−1.12; 0.41]
Network estimate			−0.20	[−0.54; 0.15]
**RMMR_DIA vs UC**				
Direct estimate	1	0.31	1.02	[0.35; 1.69]
Indirect estimate			0.67	[0.23; 1.12]
Network estimate			0.78	[0.41; 1.15]
**RMMR_DIA + MET vs UC**				
Direct estimate	3	0.59	1.16	[0.75; 1.58]
Indirect estimate			0.71	[0.22; 1.20]
Network estimate			0.98	[0.66; 1.29]
**RUMR_DIA vs UC**				
Direct estimate	2	0.89	−0.14	[−0.57; 0.28]
Indirect estimate			1.22	[−0.01; 2.44]
Network estimate			−0.00	[−0.40; 0.40]

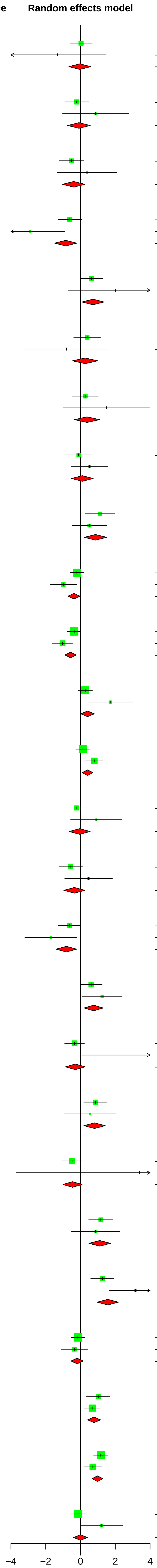

Supplementary figure S5

Comparison	Number of Studies	Direct Evidence	SMD	95%CI
**MET vs NMES**				
Direct estimate	1	0.59	0.19	[−1.60; 1.97]
Indirect estimate			−0.77	[−2.90; 1.35]
Network estimate			−0.21	[−1.57; 1.16]
**MET vs NMES + MET**				
Direct estimate	1	0.90	−0.11	[−1.89; 1.67]
Indirect estimate			−2.08	[−7.54; 3.37]
Network estimate			−0.30	[−2.00; 1.39]
**MET vs RMMR**				
Direct estimate	1	0.89	0.90	[−0.86; 2.66]
Indirect estimate			2.59	[−2.48; 7.65]
Network estimate			1.08	[−0.58; 2.74]
**MET vs RMMR + MET**				
Direct estimate	1	0.70	−0.18	[−1.92; 1.56]
Indirect estimate			1.49	[−1.17; 4.15]
Network estimate			0.32	[−1.13; 1.77]
**MET vs UC**				
Direct estimate	1	0.56	0.92	[−0.85; 2.69]
Indirect estimate			1.04	[−0.94; 3.02]
Network estimate			0.97	[−0.35; 2.29]
**NMES vs NMES + MET**				
Direct estimate	1	0.89	−0.30	[−2.11; 1.52]
Indirect estimate			1.61	[−3.63; 6.85]
Network estimate			−0.09	[−1.81; 1.62]
**NMES vs RET**				
Direct estimate	1	0.63	0.00	[−1.66; 1.66]
Indirect estimate			−2.01	[−4.16; 0.13]
Network estimate			−0.75	[−2.06; 0.56]
**NMES vs UC**				
Direct estimate	1	0.50	0.76	[−0.91; 2.44]
Indirect estimate			1.60	[−0.08; 3.29]
Network estimate			1.18	[0.00; 2.37]
**RET vs UC**				
Direct estimate	1	0.63	2.68	[1.03; 4.33]
Indirect estimate			0.66	[−1.49; 2.82]
Network estimate			1.93	[0.62; 3.24]
**RMMR vs RMMR + MET**				
Direct estimate	1	0.84	−1.08	[−2.83; 0.67]
Indirect estimate			0.98	[−3.09; 5.05]
Network estimate			−0.76	[−2.37; 0.85]
**RMMR vs UC**				
Direct estimate	1	0.81	0.02	[−1.71; 1.75]
Indirect estimate			−0.66	[−4.24; 2.92]
Network estimate			−0.11	[−1.66; 1.45]
**RMMR + MET vs UC**				
Direct estimate	2	0.95	0.57	[−0.60; 1.73]
Indirect estimate			2.40	[−2.81; 7.62]
Network estimate			0.65	[−0.48; 1.79]

Random effects model

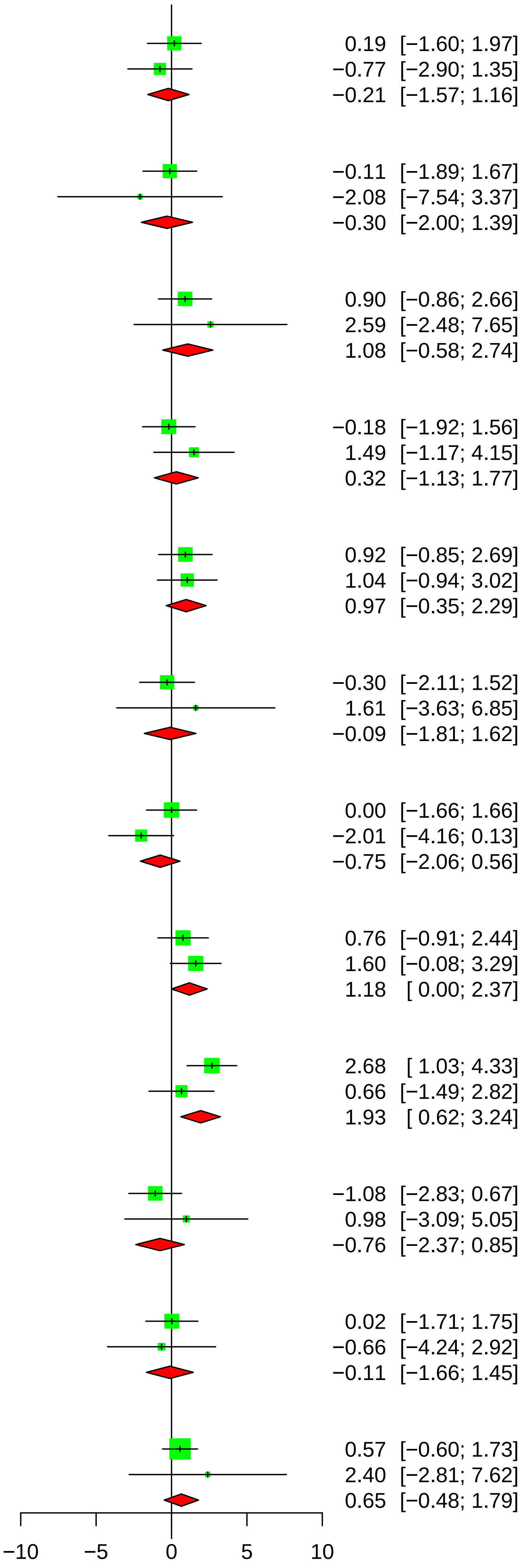

Supplementary figure S6

Comparison	Number of Studies	Direct Evidence	SMD	95%CI
**AET vs RET**				
Direct estimate	1	0.81	0.41	[−0.04; 0.85]
Indirect estimate			0.36	[−0.57; 1.29]
Network estimate			0.40	[0.00; 0.80]
**AET vs UC**				
Direct estimate	3	0.98	0.47	[0.15; 0.80]
Indirect estimate			−0.38	[−2.79; 2.04]
Network estimate			0.46	[0.14; 0.78]
**DIA + MET vs MET**				
Direct estimate	1	0.31	1.12	[0.21; 2.03]
Indirect estimate			0.13	[−0.47; 0.74]
Network estimate			0.43	[−0.07; 0.94]
**DIA + MET vs UC**				
Direct estimate	2	0.91	0.36	[0.02; 0.69]
Indirect estimate			1.34	[0.30; 2.38]
Network estimate			0.45	[0.13; 0.77]
**MET vs UC**				
Direct estimate	1	0.79	0.22	[−0.28; 0.73]
Indirect estimate			−0.76	[−1.73; 0.21]
Network estimate			0.01	[−0.43; 0.46]
**NMES vs RET**				
Direct estimate	1	0.57	0.38	[−0.34; 1.10]
Indirect estimate			0.00	[−0.83; 0.83]
Network estimate			0.22	[−0.32; 0.76]
**NMES vs UC**				
Direct estimate	1	0.56	0.11	[−0.61; 0.83]
Indirect estimate			0.49	[−0.33; 1.32]
Network estimate			0.28	[−0.26; 0.82]
**RET vs UC**				
Direct estimate	1	0.78	0.15	[−0.27; 0.57]
Indirect estimate			−0.25	[−1.05; 0.55]
Network estimate			0.06	[−0.31; 0.43]

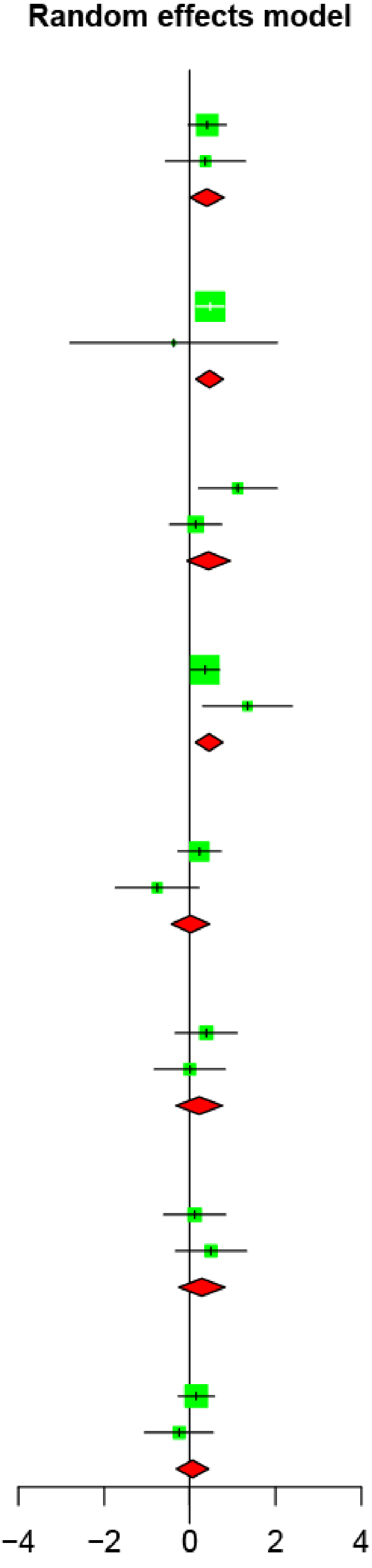

Supplementary figure S7

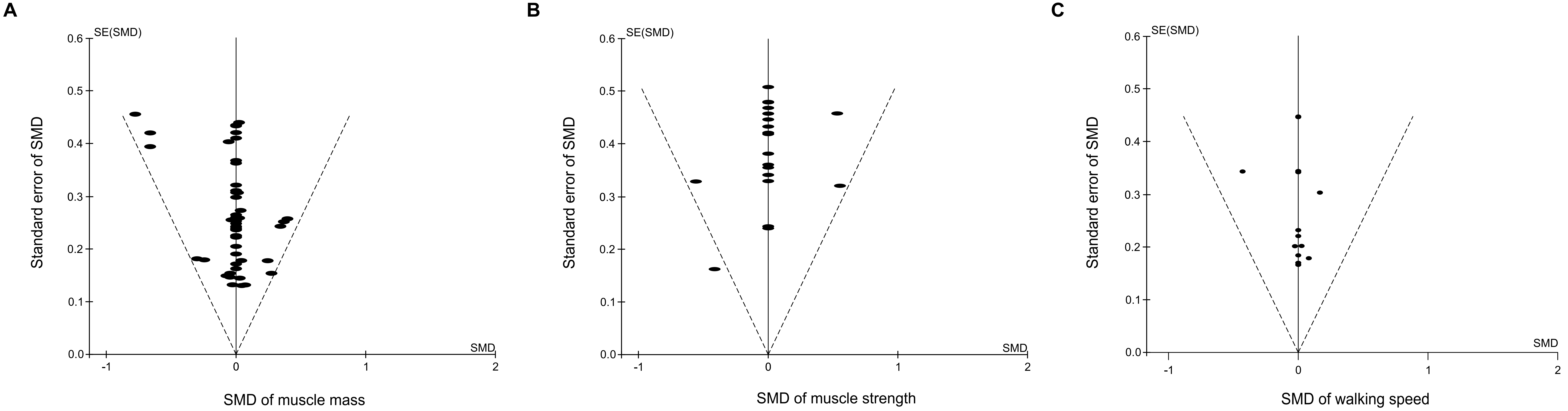

Figure S7. Funnel plots of the intervention effects for (A) muscle mass, (B) muscle strength, and (C) walking speed. Each circle denotes an independent comparison, with the X-axis representing a standard mean difference (SMD) over control comparisons and the Y-axis depicting the standard error (SE) of SMD.
